# Cellulose Acetate Nanoparticles as Eco-Friendly Photosensitizers for Antimicrobial Photodynamic Inactivation

**DOI:** 10.1021/acsomega.6c04142

**Published:** 2026-07-07

**Authors:** Raphael S. Flores, Gabriella Miessi, Priscila S. Cavalheri, Emmanuel S. C. Miguel, Regiane G. Lima, Samuel L. Oliveira, Anderson R. L. Caires

**Affiliations:** Optics and Photonics Group, Institute of Physics, Federal University of Mato Grosso do Sul, P.O. Box 549, Campo Grande, Mato Grosso do Sul 79070-900, Brazil

## Abstract

Antimicrobial resistance has emerged as a critical global health issue, demanding sustainable alternatives to conventional antibiotics. This study aimed to develop and evaluate cellulose acetate nanoparticles (NPs-CA) as eco-friendly photosensitizers for antimicrobial photodynamic inactivation (aPDI). NPs-CA were synthesized via nanoprecipitation, producing spherical and monodisperse nanoparticles with a mean hydrodynamic diameter of 268 ± 8 nm. The particles’ photophysical and morphological properties were characterized by UV–vis, SEM, and DLS. ROS generation was assessed using dihydroethidium (DHE) assays. Acute toxicity was assessed using *Artemia* sp. assays. The nanoparticles exhibited visible light absorption at 450 nm and generated ROS upon irradiation. In vitro assays demonstrated a >3 log_10_ CFU reduction of *Escherichia coli* at 500 ppm and complete photoinactivation at 1000 ppm under blue-light irradiation. *Staphylococcus aureus* exhibited low sensitivity, with no significant photodynamic effect observed. Toxicity tests indicated an LC_50_ of 320.56 ppm and a TU < 0.4, classifying the system as nontoxic. NPs-CA are light-activated photosensitizers for *E. coli* inactivation, with confirmed safety in ecotoxicological assays. These findings support the use of cellulose-based nanomaterials as sustainable and biocompatible platforms for antimicrobial photodynamic therapy, particularly against multidrug-resistant Gram-negative bacteria.

## Graphical abstract

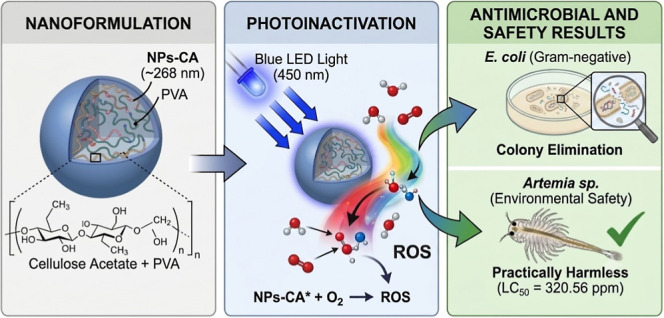

## 1 Introduction

Bacterial infections have challenged humanity throughout history, causing devastating pandemics like the Black Death (14th century), cholera (19th century), and ongoing tuberculosis.^1–3^ The discovery of antibiotics initially appeared to solve this problem, but their overuse has accelerated drug-resistant strains, returning us to a preantibiotic era.^4^ Without intervention, antimicrobial-resistant pathogens could cause over 10 million annual deaths by 2050, with a projected $100 trillion cumulative economic impact.^5^ Antibiotic resistance occurs through four primary mechanisms: reduced outer membrane permeability, enzymatic antibiotic inactivation, antibiotic efflux pumps, and drug-target modification. These processes collectively decrease intracellular antibiotic concentrations or reinforce cellular structures.^6^ Beyond future projections, antimicrobial-resistant bacteria already pose critical challenges to global healthcare systems. Notably, Gram-negative *Escherichia coli*, capable of causing gastrointestinal infections,^7^ includes resistant variants like mcr-1-positive strains that confer colistin resistance, jeopardizing last-resort antibiotic therapies.^8^ Equally concerning is Gram-positive *Staphylococcus aureus*, responsible for pneumonia and skin infections,^9^ with methicillin-resistant strains (MRSA) showing 67% higher mortality than susceptible counterparts.^10^

Photodynamic inactivation (PDI) has emerged as a promising approach for bacterial infections.^11,12^ The process requires three components: a photosensitizer (PS), molecular oxygen (O_2_), and light. The excited PS catalyzes reactions with O_2_, generating reactive oxygen species (ROS) that damage microbial cells.^13^ ROS production can occur via two mechanisms: Type I (electron transfer producing free radicals/peroxides) and Type II (energy transfer forming singlet oxygen).^14^ The key advantage of PDI as an alternative to antibiotics lies in its multisite action. While antibiotics commonly act on specific bacterial cellular sites, internalized PS generate ROS simultaneously at multiple locations, inducing great oxidative stress. Recent works demonstrated that PDI can similarly inactivate multidrug-resistant and antibiotic-susceptible bacterial strains.^15,16^ Additionally, the multisite mechanism of PDI significantly impedes the development of mutation-driven resistance.^17^

Despite the growing number of studies exploring antimicrobial photodynamic inactivation (aPDI), most reported systems rely on classical organic dyes, metallic nanoparticles, or conjugated polymers as photosensitizers. While effective, these materials often have drawbacks such as photobleaching, cytotoxicity, environmental persistence, or the need for complex synthesis routes.^11,17^ In this context, the development of sustainable, metal-free, and biodegradable photosensitizing platforms remains an important challenge. Natural and semisynthetic polymers have emerged as promising alternatives; however, their direct use as photoactive systems for aPDI remains underexplored.

Cellulose, the world’s most abundant natural biopolymer (40–50% of Earth’s biomass), is a renewable, biodegradable, and nontoxic organic polymer derived from plants, bacteria, and tunicates.^20^ Its derivative, cellulose acetate (CA), exhibits biocompatibility, biodegradability, and mechanical strength, enabling processing into films, fibers, and membranes for industrial applications.^21,22^ Recent research has enhanced CA properties by incorporating bioactive nanocomposites,^23^ antimicrobial essential oil nanocapsules,^24^ biomedical packaging materials,^25^ and drug-delivery nanoparticles.^26^ The functional groups (hydroxyl, carboxyl, and ether) on its backbone facilitate ionic interactions, making CA an excellent nanoreactor for catalytic nanoparticles.^27^ Notably, demonstrating minimal environmental toxicity, with no phytotoxic, cytotoxic, or genotoxic effects in *Allium cepa* assays.^28^ Recently, *Artemia salina* has been adopted as a biological model in nanotoxicology because its assays are simple, fast, and cost-effective.^29^

Although cellulose acetate is not a classical chromophoric material, recent studies have demonstrated that polymeric nanostructures can act as indirect photosensitizers through light-induced charge separation, defect states, or interfacial oxygen activation.^3,30^ Confinement effects, surface hydroxyl groups, and polymer–solvent interactions present in cellulose-based nanoparticles may facilitate ROS generation under visible light, even in the absence of conjugated π-systems.

In this context, the current study investigates cellulose acetate nanoparticles (NPs-CA) synthesized via nanoprecipitation as a sustainable, metal-free photosensitizing platform for antimicrobial photodynamic inactivation (aPDI). Beyond conventional carrier applications, this work explores the intrinsic photoactivity of CA-based nanostructures, investigating their physicochemical properties, ROS generation capability, antimicrobial performance against Gram-negative and Gram-positive bacteria, and environmental safety assessed through *Artemia* sp. assays. By doing so, this study aims to advance cellulose-derived nanomaterials from passive carriers to active photodynamic agents.

## 2 Materials and Methods

### 2.1 Materials

Cellulose acetate (CA) with a molecular weight approximately equal to 50,000 g/mol, poly­(vinyl alcohol) (PVA) with a molecular weight range of 30,000–70,000 g·mol^–1^, and tetrahydrofuran (THF) were acquired from Sigma-Aldrich (St. Louis, MO, USA). Plate Count Agar (PCA) and Brain-Heart Infusion Broth (BHI), used for cultivation and assays, were obtained from KASVI (São José dos Pinhais, PR, BR). Finally, microbiological assays were conducted using *S. aureus* ATCC 25923 and *E. coli* ATCC 25922.

### 2.2 Nanoparticles Synthesis

The synthesis of the NPs was carried out by the nanoprecipitation method.^28,31^ First, an organic solution was prepared by adding 40 mg of CA to 4 mL of THF. An aqueous solution was also prepared by dissolving 13.75 mg of PVA in 2.5 mL of distilled water. Then, the organic solution was added dropwise to the aqueous solution under magnetic stirring. The solutions were protected from light and stirred continuously at 80 rpm for 24 h at 25 °C to ensure complete solvent evaporation. Afterward, the volume of the solution was adjusted to 10 mL by adding distilled water to achieve a final concentration of 4 mg mL^–1^ of CA. The overall synthesis process is illustrated in Figure 1.

**Figure 1:**
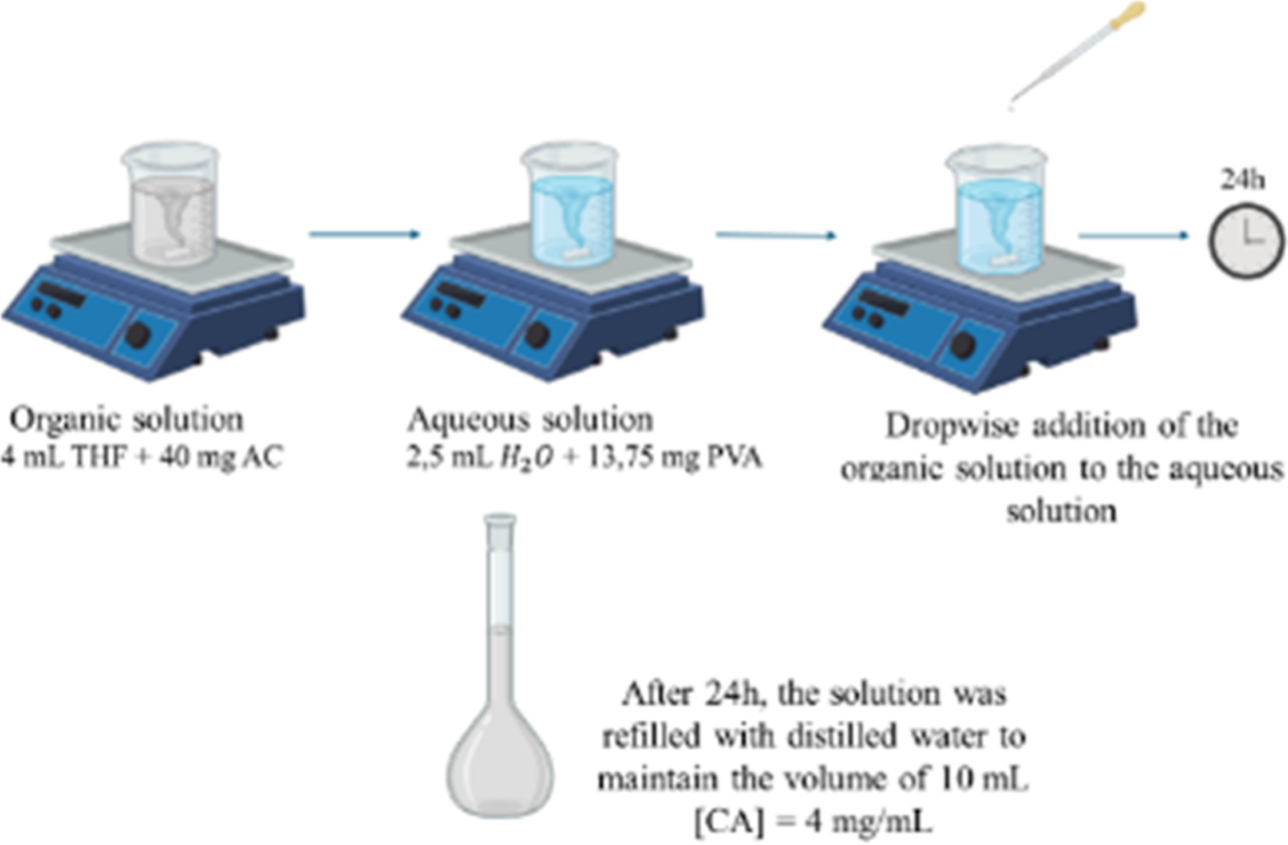
Schematic representation of the nanoprecipitation method used for the synthesis of cellulose acetate nanoparticles (NPs-CA). An organic solution (4 mL THF + 40 mg CA) was added dropwise to an aqueous solution (2.5 mL H_2_O + 13.75 mg PVA) under magnetic stirring. The mixture was stirred at room temperature for 24 h to allow solvent evaporation, and the final volume was adjusted to 10 mL with distilled water, yielding a CA concentration of 4 mg·mL^–1^. Created by the authors using BioRender.com.

### 2.3 UV–Vis Absorption Characterization

A UV–vis spectrophotometer (LAMBDA 265 UV/vis) was used to measure the absorbance of the NPs-CA. For the measurement, a 200 μL aliquot of the NPs-CA solution (4 mg·mL^–1^) was diluted with 1800 μL of distilled water. The diluted sample was placed in a 1 cm quartz cuvette with four polished sides. A baseline was established using distilled water before the absorption spectrum was recorded in the 200–700 nm range.

### 2.4 Dynamic Light Scattering Analysis

The hydrodynamic diameter distribution, polydispersity index (PDI), and zeta potential (ζ) of the NPs-CA were determined using a Zetasizer Nano-ZS ZEN 3600 (Malvern). For these measurements, a solution of NPs-CA at a concentration of 4 mg/mL was diluted in Milli-Q water. The analyses were conducted at a constant temperature of 25 °C and a constant pH, using an electrophoresis cuvette (DTS1070).

### 2.5 Scanning Electron Microscope Images

SEM analysis was performed using a JEOL JSM-6380 LV microscope (JEOL, Akishima, Tokyo, Japan). Images of the NPs-CA were acquired after depositing the nanoparticles onto a metal stub, followed by drying and sputtering with a thin conductive gold layer. Morphological analysis was conducted on images at 20,000× and 50,000× magnification, and nanoparticle size distribution was determined by measuring 100 randomly selected particles using ImageJ software.

### 2.6 Fourier Transform Infrared Spectroscopy Analysis

NPs-CA and their pure components were analyzed by FTIR spectroscopy in an Agilent Cary 630 FTIR spectrometer (California, USA), equipped with an attenuated total reflectance (ATR) accessory featuring a germanium crystal. Before analysis, the nanoparticles were centrifuged and oven-dried to obtain powdered samples. Ten scans were recorded in the spectral range of 4000 to 500 cm^–1^ with a resolution of 0.5 cm^–1^.

### 2.7 Thermal Analysis

Thermogravimetric analysis (TGA) and differential scanning calorimetry (DSC) were used to study the phase transitions of NPs-CA and compare them with those of pure AC polymer. Analyses were performed using a NETZSCH STA 449 F3 Jupiter (Selb, Germany) instrument, with the temperature ranging from 20 to 600 °C at a heating rate of 15 °C·min^–1^.

### 2.8 Photoinactivation Assay

The bacterial strains utilized for the photoinactivation assay were *E. coli* (ATCC 25922) and *S. aureus* (ATCC 25923). These strains were preserved at 2 °C in Müller-Hinton broth (Sigma-Aldrich, St. Louis, USA) supplemented with 20% glycerol to maintain viability. To prepare the working bacterial suspensions, 40 μL of each bacterial stock was inoculated into 20 mL of Brain Heart Infusion (BHI) medium. This mixture was then incubated at 37 °C with agitation for 24 h to facilitate bacterial growth. The concentration of the resulting bacterial suspension was then standardized to a 1.0 McFarland scale by adding phosphate-buffered saline (PBS). This standardization was achieved by adjusting the suspension until its absorbance at 625 nm reached approximately 0.14, measured in an Agilent BioTek Synergy H1Multimode Reader (Santa Clara, CA, USA). Various concentrations of NPs-CA solutions were prepared by diluting the 4 mg mL^–1^ stock solution. Then, 500 μL of each NPs-CA solution was mixed with 500 μL of the bacteria-saline suspension, resulting in final concentrations of 0.5 McFarland for the bacteria and 100, 250, 500, and 1000 ppm for NPs-CA. A negative control was also prepared by mixing 500 μL of the bacteria-saline suspension with 500 μL of PBS. Samples were divided in two groups: irradiated and nonirradiated. Both groups were kept in the dark for 60 min to allow nanoparticle internalization by the bacteria. After this period, 200 μL of each concentration from the irradiated group was transferred to a 96-well plate and exposed to blue LED light at 450 nm (28 mW cm^–2^) for 90 min, while the nonirradiated group remained in darkness. Finally, 200 μL from each concentration of both groups were diluted in PBS to a 1:32 ratio and plated on plate count agar (PCA) using the spread plate method in a 96-well format. Colony-forming units (CFUs) were counted after 24 h of incubation at 37 °C. All measurements were performed in duplicate, and the entire experiment was repeated three times to ensure statistical reliability. For data analysis, raw CFU counts were logarithmically transformed (log_10_) to meet the assumptions for parametric statistical tests. Standard deviation was calculated for each experimental condition across the independent replicates, and statistical significance for observed differences between experimental groups was determined using the *t*-Student test (p-value <0.05).

### 2.9 Reactive Oxygen Species Determination

The generation of reactive oxygen species (ROS) was quantified according to an adapted protocol based on the method of Caires et al. [29]. A volume of 70 μL of 5 mM dihydroethidium (DHE) was introduced to 2 mL of an aqueous suspension of NPs-CA at a concentration of 500 ppm. After 10 min in the dark, the mixture was irradiated with a blue LED (450 nm) at an irradiance of 28 mW cm^–2^ for 25 min. Within this 35 min, fluorescence emission spectra were acquired at 1 min intervals across the 520–700 nm range, with an excitation wavelength of 500 nm, using a Sinco FluoroMate FS-2 spectrofluorometer (Seoul, Republic of Korea).

### 2.10 Acute Toxicity Assay in *Artemia* sp

The acute toxicity assay was performed according to^32,33^ and the ABNT NBR 16530:2016 Brazilian standard.^34^ The results were expressed in median lethal concentration (LC_50_), defined as the concentration at which 50% of exposed organisms exhibit mortality.^35^ For cyst hatching, a synthetic seawater solution at 36 g L^–1^ and pH 8–9 was aerated for 48 h. After hatching, 10 individuals were added to each 10 mL beaker containing 3 mL sample. The temperature was maintained at 25 °C, with a 16 h light:8 h dark photoperiod in a static system. The LC_50_ was calculated for dilutions of 1000, 750, 500, 250, 100, 50, 5, and 1 ppm. The pH of the samples was adjusted to 7.0 with 1 mol L^–1^ NaOH solution. The saline solution was used as a negative control and for sample dilution. Potassium dichromate at 1% was used as a positive control. The assays were performed in triplicate. The LC_50_ values were calculated from the linear regression of the ratio between the percentage of dead (immobilized) nauplii and the sample concentration. A nauplius was considered immobile when it could not move its appendages or barycenter position for 10 s. For this purpose, PROBIT analysis was conducted using StatPlus/mac, AnalystSoft, v8.^36,37^

## 3 Results

### 3.1 Nanoparticles Characterization

Figure 2 shows a typical UV–vis absorption spectrum of NPs-CA. The spectrum exhibits a monotonically decreasing absorbance from 250 to 700 nm, with no clear absorption peaks. This pattern is consistent with the nonconjugated nature of the polymer backbone, which lacks strong chromophoric groups.^30^ The absorbance profile of SEM micrograph analysis of the NPs-CA (Figure 3b) revealed a mean particle diameter of 206 ± 7 nm. In contrast, dynamic light scattering (DLS) measurements (Figure 3a) showed a larger mean hydrodynamic diameter of 268 ± 8 nm, as expected due to the contribution of hydration layers surrounding the nanoparticles in solution. Additionally, DLS analysis showed a PDI of 0.15 ± 0.02, indicating monodisperse behavior of NPs-CA in aqueous solution, as PDIs between 0.1 and 0.7 are typical of monodisperse systems.^37^ The zeta potential of NPs-CA was −8.75 ± 0.23 mV, a moderately negative value that suggests limited electrostatic stabilization but adequate colloidal stability due to steric effects, consistent with cellulose-based systems.^39^ The size of cellulose acetate (CA)-based nanoparticles is highly affected by the synthesis method. For example, CA nanocarriers produced via supercritical electrospray-assisted techniques exhibited diameters ranging from 266 to 343 nm, depending on the polymer concentration during processing.^38^ In comparison, NPs-CA synthesized via a nanoemulsion method with dichloromethane as the organic solvent displayed significantly larger average sizes, reaching up to 508 nm [39].

**Figure 2:**
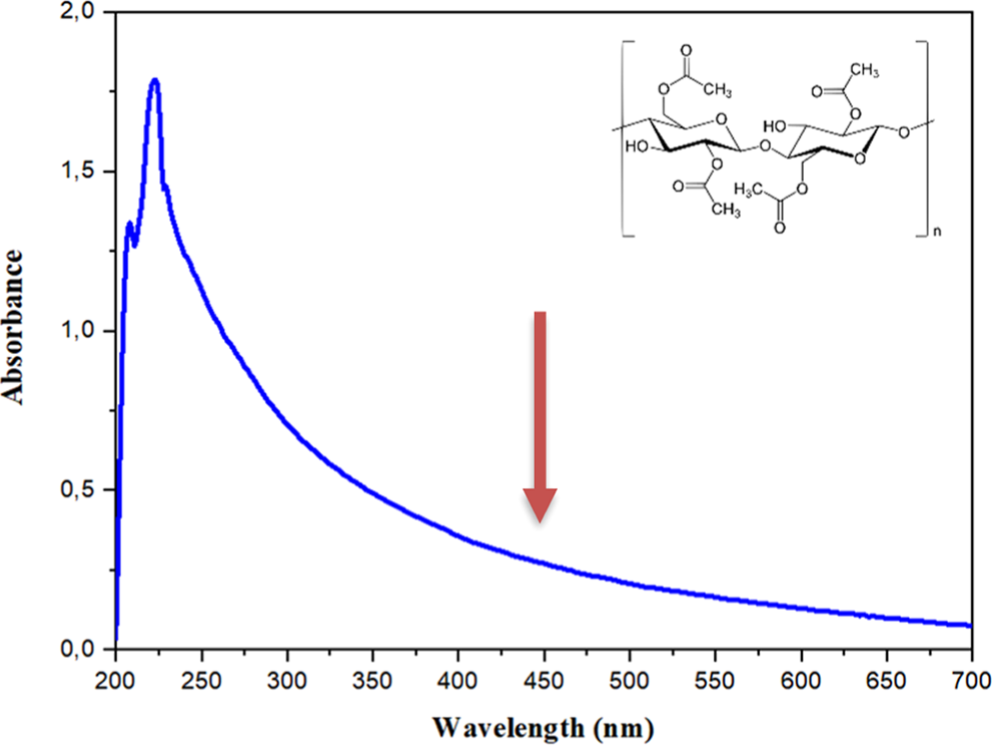
UV–vis absorbance spectrum of NPs-CA solution at 100 ppm, with an arrow indicating the wavelength at 450 nm corresponding to blue light irradiation. The inset shows the chemical structure of cellulose acetate.

**Figure 3:**
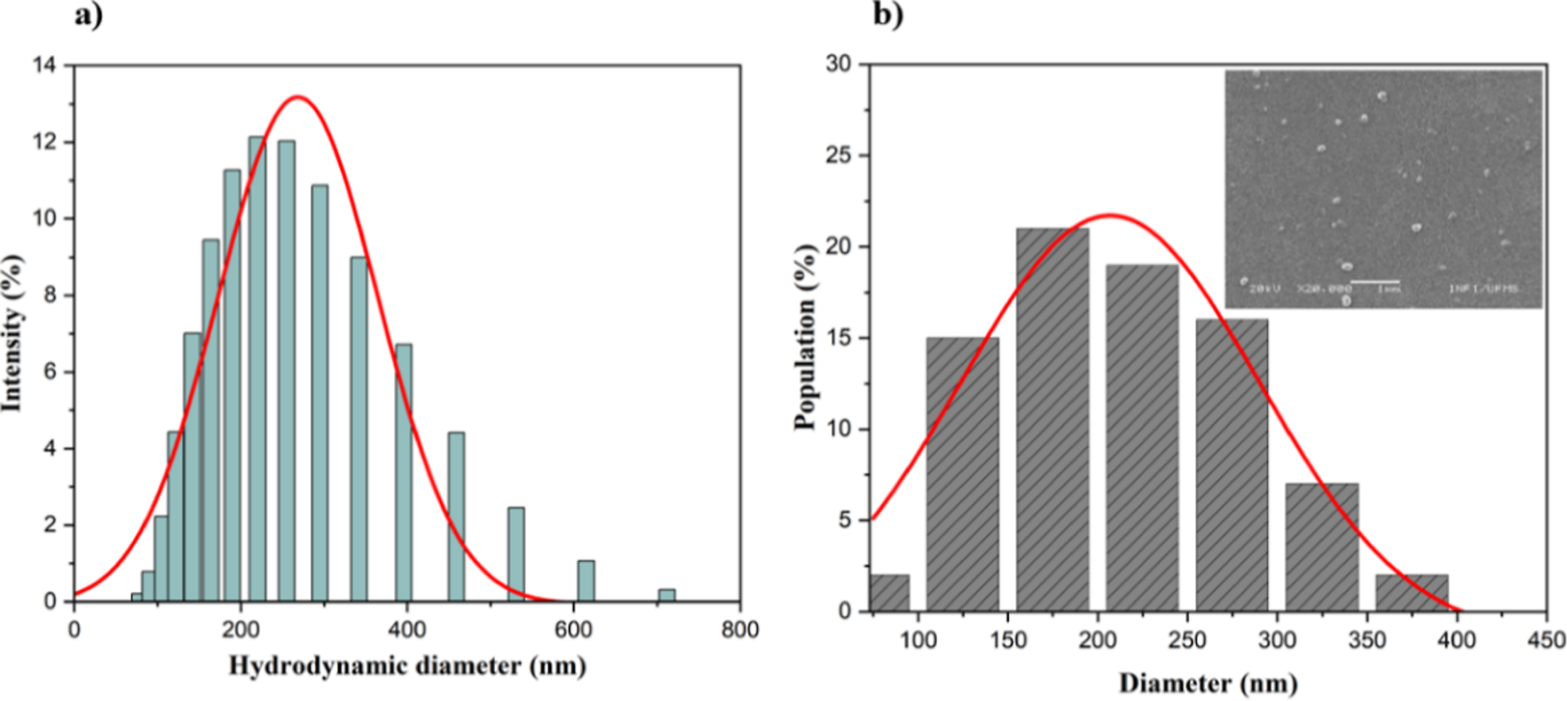
Diameter distribution of NPs-CA measured by (a) DLS and (b) SEM. Inset: representative SEM image of NPs-CA.

Figure 4 shows the FTIR spectra of the NPs-CA compared with their precursor materials, CA and PVA. This analysis confirms the chemical composition of the nanoparticles and provides insight into the molecular interactions occurring during the nanoprecipitation process. The NPs-CA spectrum preserves the main vibrational features of the CA polymer backbone, indicating that its structural integrity is maintained after synthesis. The characteristic C=O stretching band at 1738 cm^–1^, associated with ester groups in CA, and the C–O stretching of acetyl groups at 1220 cm^–1^ are clearly observed. Subtle spectral variations, particularly slight shifts in the 1500–1000 cm^–1^ region, provide valuable insight into the nanoparticle formation mechanism by indicating significant intermolecular interactions between CA and PVA. In addition, a pronounced reduction in the –OH stretching band in the NPs-CA spectrum, compared to pure PVA, suggests the establishment of intermolecular hydrogen bonding between the hydroxyl groups of PVA and the carbonyl or ether oxygen atoms in the CA matrix. These interactions play a critical role in stabilizing the nanoparticle surface during solvent evaporation, ultimately enabling the formation of a cohesive and monodisperse system.^38,39^

**Figure 4:**
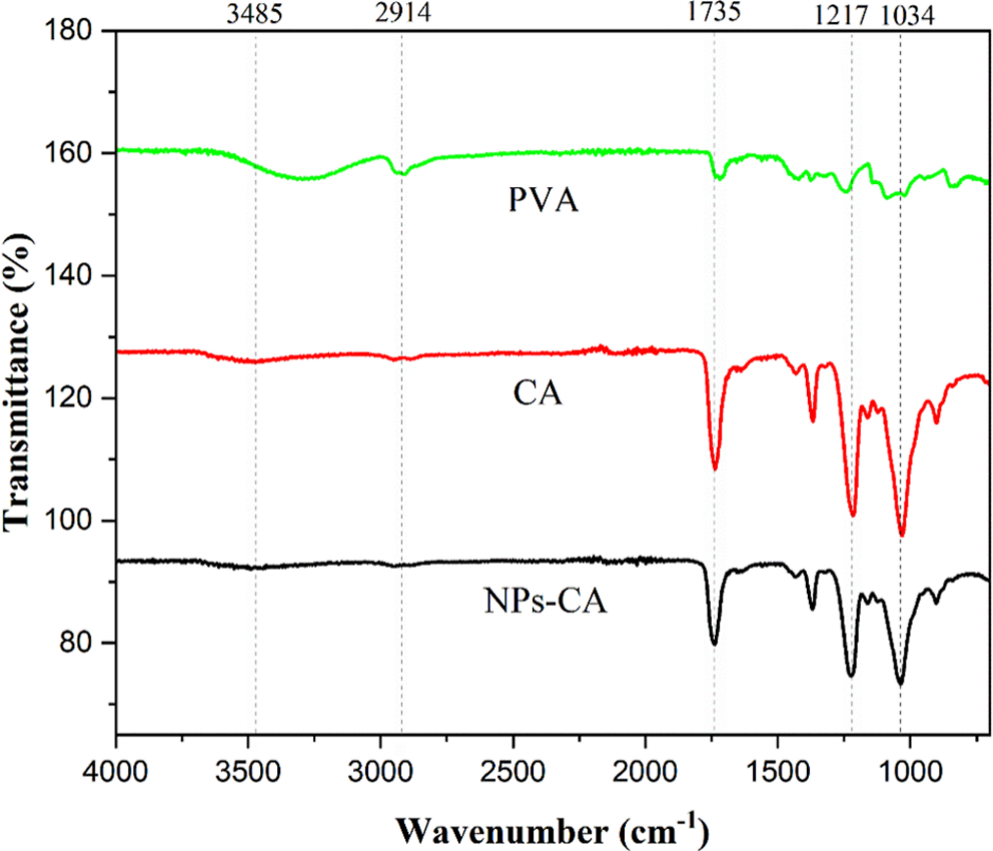
FTIR spectra of NPs-CA and their individual constituents.

The thermal stability and phase transitions of the NPs-CA were evaluated using Thermogravimetric Analysis (TGA) and Differential Scanning Calorimetry (DSC), as shown in Figure 5. The TGA curve revealed a notable mass loss between 300–400 °C, characteristic of CA thermal degradation.^40^ Apart from the dehydration-related loss of around 100 °C, absent in dried samples, the NPs-CA thermal profile closely matches that of pure CA. Similarly, the DSC curve of NPs-CA displays an endothermic event near 200 °C, linked to polymer crystallization, consistent with the thermal behavior of CA.^40^ These data confirm that NPs-CA act as a thermally stable carrier, preserving the intrinsic thermal properties of CA and highlighting their suitability as a durable and biocompatible platform for light-activated antimicrobial inactivation.

**Figure 5:**
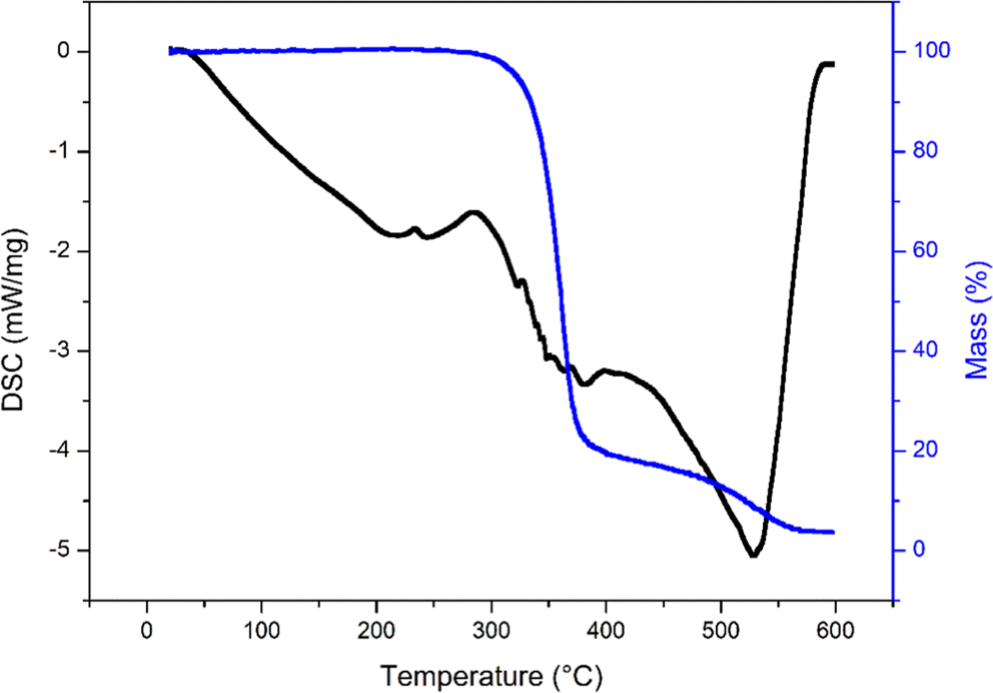
Differential scanning calorimetry (DSC) and thermogravimetric analysis (TGA) curves of NPs-CA.

### 3.2 Photoinactivation Assay

The antimicrobial photoinactivation assay was conducted to assess the photosensitizing potential of NPs-CA (Figure 6). For *S. aureus*, a modest reduction in CFU was observed across all irradiated treatments, including the negative control, indicating that blue light alone (in the absence of NPs-CA) inhibited bacterial growth—a known effect for this species.^39,42^ However, this reduction was less than 1 log_10_ CFU and therefore cannot be classified as bactericidal.^43^ In contrast, NPs-CA demonstrated significant photobactericidal activity against *E. coli*, achieving >3 log_10_ CFU reduction at 500 and 1000 ppm. At 1000 ppm, complete photoinactivation was achieved, with no detectable bacterial growth. Statistical analysis comparing the light and dark groups confirmed the significance of these findings (*p* < 0.05), establishing that the observed effects were indeed due to light exposure and not any chemical effect in the dark Table 1.

**Figure 6:**
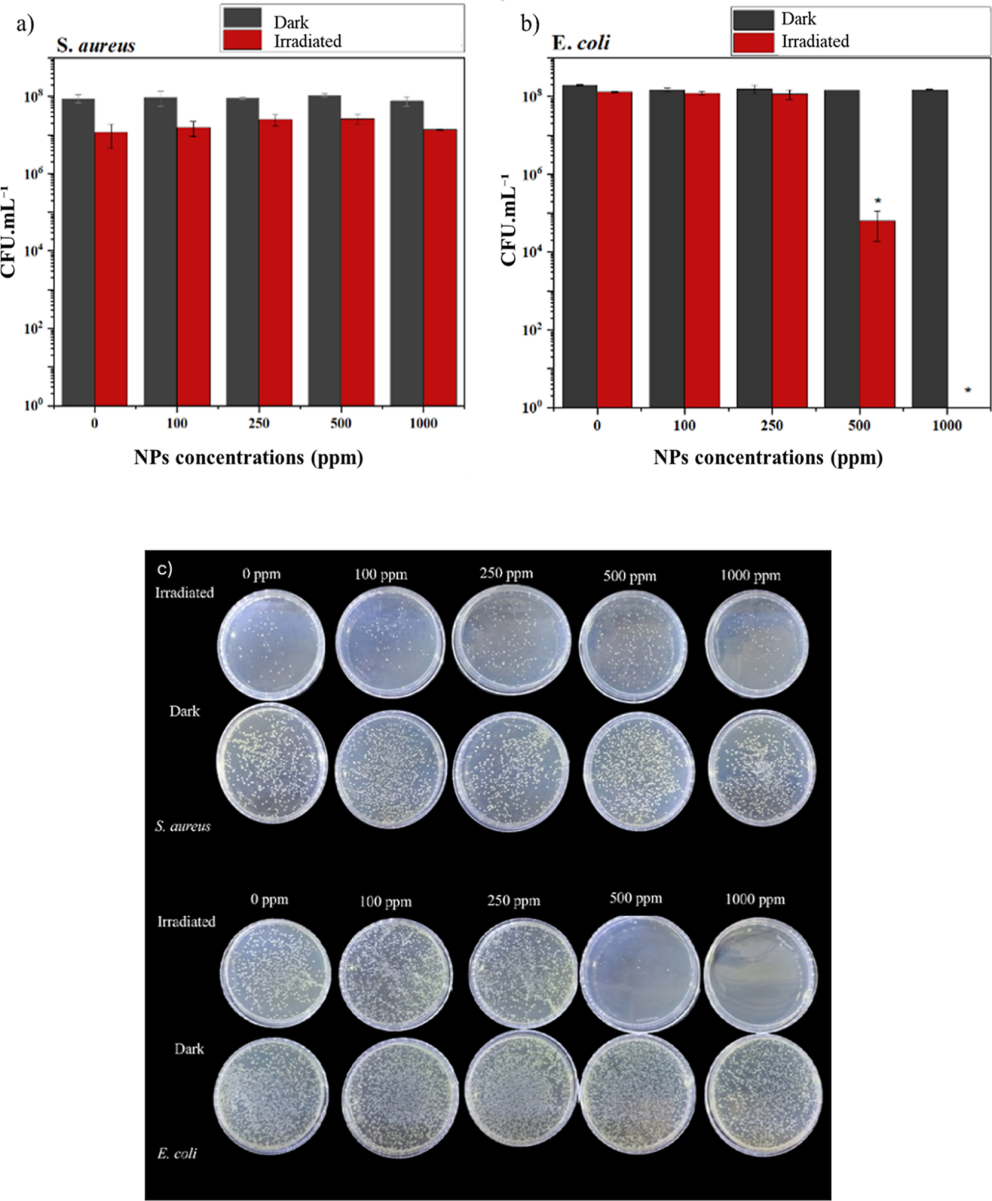
Mean CFU·mL^–1^ of (a) *Staphylococcus aureus* and (b) *Escherichia coli* in PDI assays following blue light irradiation (450 nm) for 1 h and 30 min. (*) indicates photobactericidal activity, defined as a reduction greater than 3 log_10_ CFU. (c) Representative images of CFU growth in Petri dishes.

**Table 1:** Characteristic Infrared Absorption Peaks of NPs-CA, CA, and PVA Samples^40,41^

NPs-CA	CA	PVA	IR Modes
3490 cm^–1^	3485 cm^–1^	3281 cm^–1^	O–H stretching
		2914 cm^–1^	CH_2_ asymmetric stretching
1738 cm^–1^	1735 cm^–1^	1720 cm^–1^	C=O stretching (ester)
1220 cm^–1^	1217 cm^–1^		Acetyl group C–O stretching
1034 cm^–1^	1034 cm^–1^	1071 cm^–1^	Primary alcohol C–O symmetric stretching

### 3.3 Reactive Oxygen Species Determination

To determine whether the photobactericidal activity of NPs-CA results from photodynamic action, the generation of ROS by NPs-CA under blue light exposure was quantified. This was achieved by monitoring the fluorescence of ethidium, formed through the reaction between ROS (produced by the interaction of photoactivated NPs-CA and molecular oxygen) and the nonfluorescent probe dihydroethidium (DHE).^44^ Figure 7 shows the fluorescence intensity at 612 nm measured over 10 min in the dark, followed by 15 min under blue light irradiation. No increase in fluorescence was observed during the dark period, indicating the absence of ROS generation. In contrast, a marked increase in fluorescence was detected upon illumination, demonstrating that NPs-CA rapidly generate ROS under light exposure. This fluorescence, observed in the 530–730 nm range, results from the formation of ethidium, a product of the reaction between ROS and the DHE probe. The time-dependent increase in fluorescence under illumination was fitted using the equation *F* = *a*(1-*e* ^–*k*_f_ *t*^), where F represents the fluorescence intensity at 612 nm, t is the illumination time, and *a* and *k*_f_ are fitting constants obtained from the curve fitting shown in the inset in Figure 7b. The parameter *k*_f_ corresponds to the product of the ROS generation rate constant (*k*_ROS_) and the DHE concentration, (i.e., *k*_f_ = *k*_ROS_[DHE]).^45^ For NPs-CA, the values of *k*_f_ and *k*_ROS_ were determined to be 3.2 × 10^–3^ s^–1^ and 18 M^–1^·s^–1^, respectively (Table 2). The obtained *k*_ROS_ value falls within the typical range observed for reaction rates involving free radicals (e.g., H_2_O_2_ and O_2_^–^) and various target molecules.^3^

**Figure 7:**
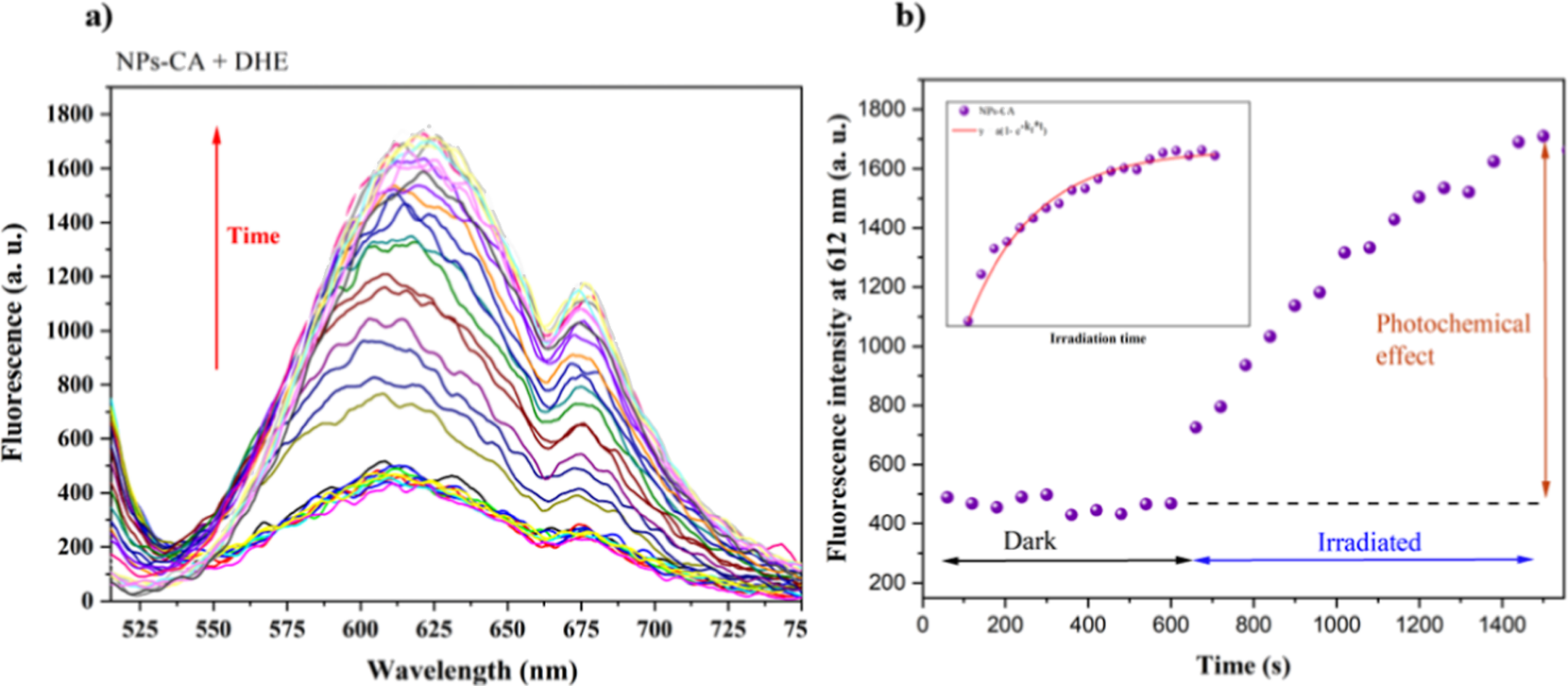
(a) Fluorescence profile of the NPs-CA + DHE solution over time. (b) Fluorescence intensity at 612 nm as a function of time, including an initial 10 min period in the dark followed by 15 min of illumination. The inset in (b) displays the fitted curve for the kinetics of ROS production under illumination (*R* ^2^ = 0.9755).

**Table 2:** ROS Production Rate of NPs-CA under Blue Light Irradiation

	*k*_f_ (10^–3^ s^–1^)	*k*_ROS_ (10^–3^ M^–1^ s^–1^)
NPs-CA	3.2 ± 0.2	18 ± 1

Additionally, control experiments were performed to confirm that the observed ROS generation is associated with the CA present in NPs-CA. As presented in the Supporting Information, Figure S1 shows that the control solution containing only DHE in aqueous medium did not exhibit any increase in fluorescence under blue light irradiation, demonstrating that neither the probe nor the irradiation conditions alone induce ROS formation. Furthermore, Figure S2 demonstrates that PVA, used solely as a stabilizing agent during nanoparticle preparation, does not exhibit absorption in the spectral region corresponding to the irradiation wavelength employed in this study. Consequently, these results exclude the contribution of residual formulation components and indicate that photodynamic activity arises predominantly from the CA structure within the nanoparticles.

### 3.4 Acute Toxicity Assay in *Artemia* sp

The toxicity assay conducted with *Artemia* sp. nauplii exposed to NPs-CA showed low observable adverse effects after 24 h of exposure across the full tested concentration range. The LC_50_ was determined by following Finney’s PROBIT regression method as presented in detail in Section S3 of the Supporting Information. As presented in Table 3, the resulting LC_50_ value was calculated as 320.56 ± 12.87 ppm. According to the toxicity scale proposed by Passino and Smith (1987),^46^ adopted by the U.S. Fish and Wildlife Service,^47^ NPs-CA fall within the “no acute toxicity” category.

**Table 3:** Acute Toxicity for *Artemia* sp. after 24 h Exposure to NPs-CA

sample	LC_50_	toxic units (TU)	toxicity
NPs-CA	320.56 ± 12.87	0.32 ± 0.01	no acute toxicity

Additionally, no observable morphological alterations, behavioral changes, or significant mortality events were observed at any of the tested concentrations within the 24 h exposure window. This observation corroborates the broader evidence indicating that cellulose-based nanomaterials generally exhibit limited ecotoxicological impact in short-term marine invertebrate assays, supporting their classification as environmentally compatible within similar exposure scenarios.^48^

## 4 Discussion

The results presented highlight the potential of NPs-CA produced through the nanoprecipitation, as an effective platform for antimicrobial photodynamic inactivation (aPDI). This method enables the formation of well-dispersed nanoparticles with a narrow size distribution (PDI ≈ 0.15), a mean hydrodynamic diameter of 268 ± 8 nm, and spherical morphology. The average dry size recorded by SEM (206 ± 7 nm) was consistent with the absence of a hydration layer.

The absorption in the visible range confirmed the photoactivation potential of NPs-CA under blue LED (about 450 nm). This phenomenon is fundamental for aPDI applications, in which light activation of the photosensitizer is required to generate cytotoxic ROS. FTIR analyses confirmed the chemical integrity of cellulose acetate within the nanostructure and revealed slight peak shifts, suggesting interactions between CA and PVA, potentially stabilizing the nanoparticle surface.

Thermal characterization showed that the nanoparticles retained the thermal degradation profile of pure cellulose acetate, including the main degradation phase between 300–400 °C and an endothermic event near 200 °C related to polymer crystallization.^40^ These findings indicate structural preservation of the biopolymer and support the use of CA as a thermally stable carrier.

Beyond confirming structural integrity, the preserved chemical structure and surface functionalities of cellulose acetate are crucial role in its photodynamic performance. The presence of residual hydroxyl and ester groups at the nanoparticle interface may facilitate oxygen adsorption and interfacial charge transfer under irradiation, contributing to ROS generation. Similar interfacial effects have been reported in polymeric and cellulose-based nanostructures, where nanoscale organization compensates for the absence of classical chromophores.^30,45^

Biological assays revealed that NPs-CA effectively induced photoinactivation of *E. coli* in a concentration-dependent manner. At 500 and 1000 ppm, a photobactericidal effect was observed (>3 log_10_ CFU reduction), with complete inactivation at 1000 ppm. The Gram-negative nature of *E. coli*, often considered more resistant to photodynamic therapy due to its complex outer membrane, makes this finding particularly relevant. In contrast, although *S. aureus* showed reduced viability under irradiation, the effect was less pronounced, and even the control group under light exhibited a reduction in CFU. This is consistent with prior reports on the sensitivity of *S. aureus* to blue light exposure alone,^11^ and highlights the need to differentiate between photodynamic action promoted by exogenous and endogenous photosensitizers.^49^

Quantitative ROS measurements using DHE confirmed that NPs-CA generate ROS under illumination. The derived reaction rate constant was within the expected range for free–radical interactions, reinforcing the oxidative mechanism of bacterial inactivation via photodynamic action. These results are consistent with previous studies on polymer-based photosensitizing systems^14,50^

Acute toxicity was assessed using *Artemia* sp. to evaluate the potential environmental impacts of NPs-CA in treatment processes. The results demonstrated that NPs-CA exhibited low acute toxicity under the tested conditions, confirming their suitability for applications in which environmental exposure may occur. By ruling out ecotoxicological effects as a contributing factor, these findings reinforce that the performance observed in the treatment process can be attributed to the intrinsic photodynamic properties of the nanoparticles rather than to nonspecific toxic action.

Compared to other cellulose-based antimicrobial systems, which frequently depend on metallic nanoparticles or encapsulated bioactive agents to achieve photodynamic or antimicrobial effects,^24,25^ the NPs-CA presented here operate as a single-component, metal-free photosensitizing platform. This simplicity reduces environmental risks, synthesis complexity, and regulatory barriers, while still providing effective photobactericidal performance under visible light.

## 5 Conclusion

The synthesized NPs-CA show promising characteristics that position them as an innovative solution for aPDI. Their small average size (268 nm), uniform dimensions, and ability to absorb visible light are critical attributes for photosensitizing applications. Furthermore, thermal and spectroscopic analyses confirmed that NPs-CA retain the properties of pure cellulose acetate, ensuring their structural biocompatibility. Biological assays, especially those against *E. coli*, revealed that NPs-CA exhibit concentration-dependent bactericidal effects, underscoring their potential as photosensitizers. The use of cellulose acetate (a naturally abundant, biodegradable, and biocompatible polymer) highlights the sustainability of this approach, aligning with the demand for environmentally responsible solutions to combat antibiotic-resistant bacteria.

## Supplementary Material



## Data Availability

Data is provided within the manuscript. Additional data is available upon request.

## Notes

ROS production under blue light irradiation (Figure S1); UV–vis absorption spectrum of PVA and emission profile of blue LED light (Figure S2); acute toxicity assay of cellulose acetate nanoparticles in Artemia sp., including mortality curve and LC50 determination by PROBIT analysis (Figures S3–S4) (PDF)
