# Supplementary material for: Cellulose Acetate Nanoparticles as Eco-Friendly Photosensitizers for Antimicrobial Photodynamic Inactivation
Source: ACS Omega. 2026 Jul 7;11(28):42742–50. doi: 10.1021/acsomega.6c04142 (PMC13393358; doi:10.1021/acsomega.6c04142)
Supplement: Supplementary file 1 [file ao6c04142_si_001.pdf]

Supporting Information

Cellulose Acetate Nanoparticles as Eco-Friendly Photosensitizers for Antimicrobial Photodynamic Inactivation

Raphael S. Flores[1], Gabriella Miessi[1], Priscila S. Cavalheri[1], Emmanuel S. C. Miguel[1], Regiane G. Lima[1], Samuel L. Oliveira[1], Anderson R. L. Caires[1*]

[1] Optics and Photonics Group, Institute of Physics, Federal University of Mato Grosso do Sul, PO Box 549, 79070-900. Campo Grande, MS, Brazil;

Keywords: Antimicrobial photodynamic inactivation; Cellulose acetate nanoparticles; Reactive oxygen species; Sustainable nanomaterials; *Escherichia coli*.

* Corresponding authors: anderson.caires@ufms.br

S1 – ROS Production under blue light irradiation

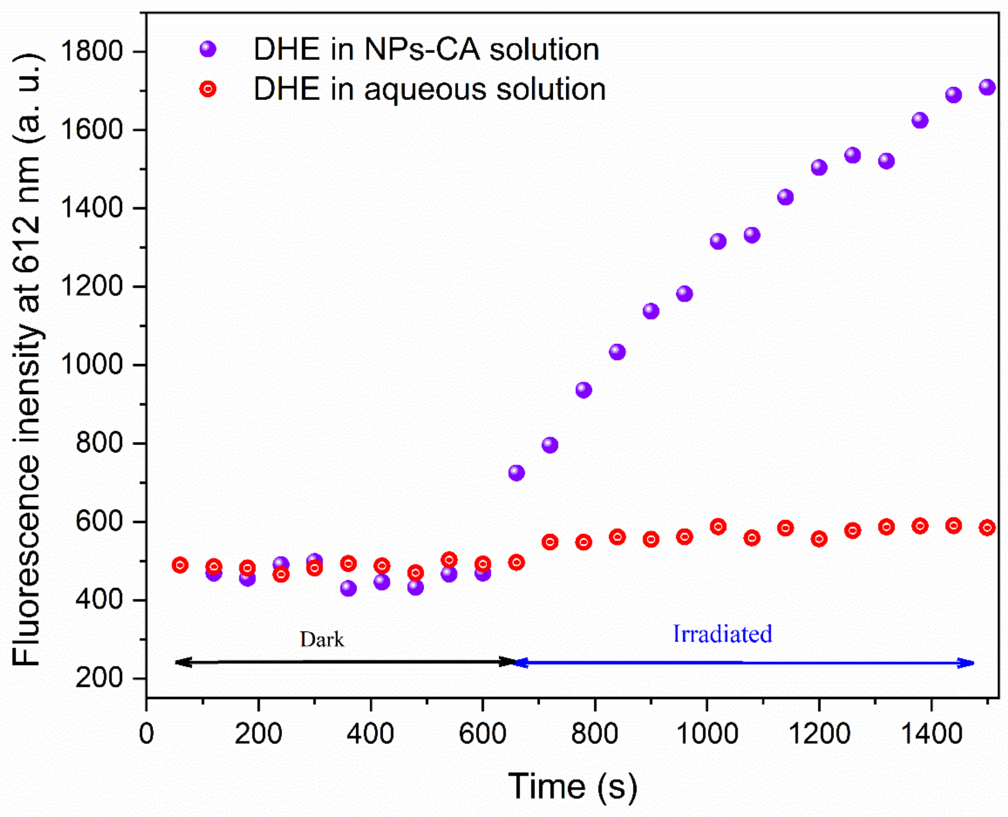

Figure S1 - Fluorescence intensity at 612 nm of the NPs-CA + DHE solution and DHE in aqueous solution as a function of time, including an initial 10-minute period in the dark followed by 15 min of illumination.

S2 – PVA absorption spectrum and emission profile of blue LED light

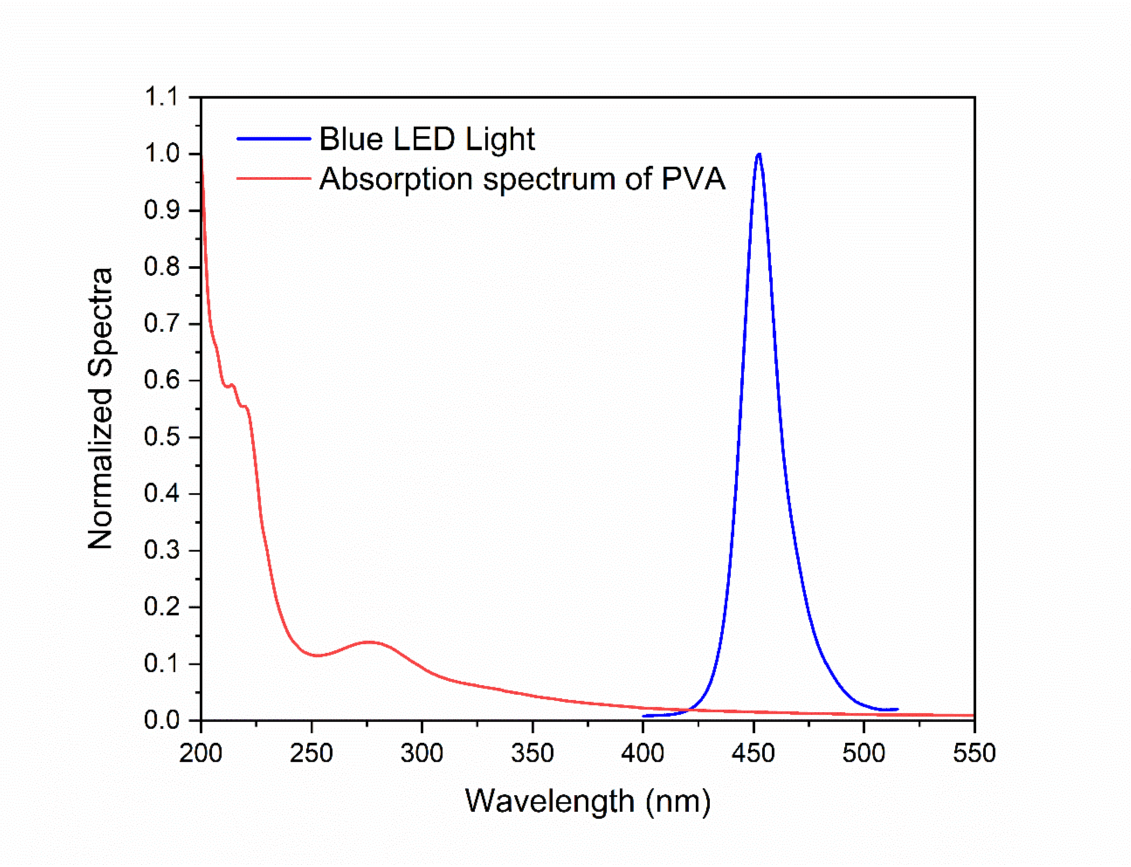

Figure S2 – UV-vis absorption spectrum of PVA and emission profile of blue LED light.

S3 – Acute toxicity assay in *Artemia sp.*

The acute toxicity assay conducted over a 24-h period demonstrated a clear dose-dependent relationship between the concentration of NPs-CA and the mortality rate of *Artemia sp.* As illustrated in Figure S3, the mortality percentage increased progressively with higher concentrations of NPs-CA. The experimental data were fitted to a dose-response curve, yielding a coefficient of determination of $R^2 = 0.9619$.

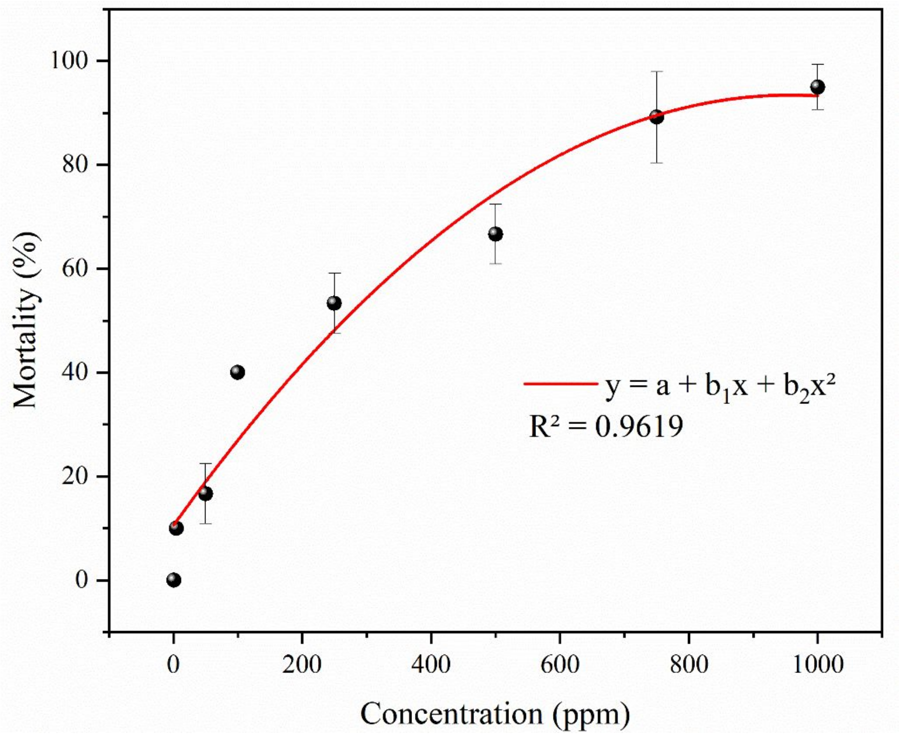

Figure S3 – Mortality (%) of *Artemia sp.* over concentration (ppm) of NPs-CA.

To accurately determine the median lethal concentration (LC50) of the CA-NPs, the mortality data obtained from the assay were subjected to PROBIT analysis using the StatPlus:mac software (AnalystSoft, v8). The PROBIT regression model transforms the sigmoidal dose-response curve into a linear relationship, allowing for a precise interpolation of the concentration required to cause 50% mortality in the test population. The PROBIT curve generated by the software is presented in Figure S4. Based on this analysis, the 24-hour LC50 value of NPs-CA for *Artemia sp.* was calculated to be 320.56 ± 12.87 ppm.

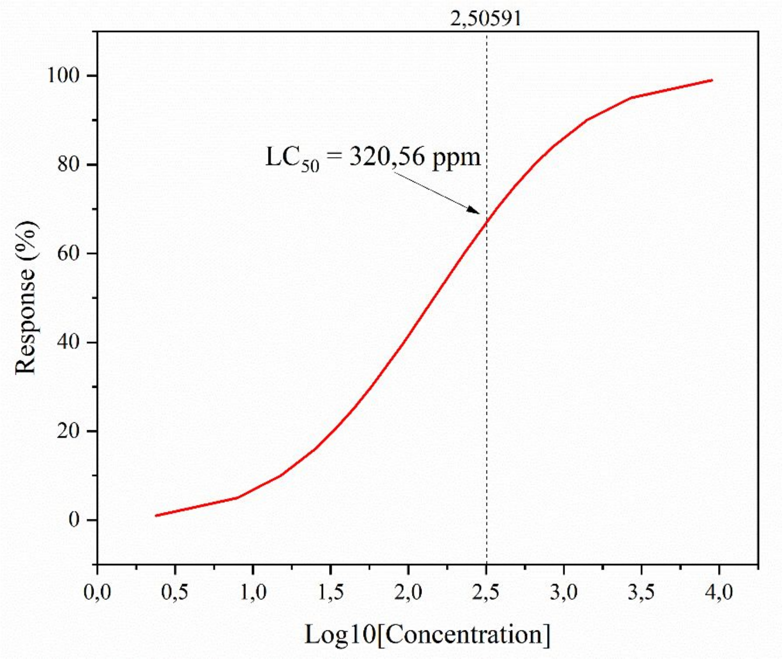

Figure S4 – PROBIT curve of Response (%) over the Log10 [concentration], with the LC50 highlighted.
